# Environment Control to Improve Recombinant Protein Yields in Plants Based on *Agrobacterium*-Mediated Transient Gene Expression

**DOI:** 10.3389/fbioe.2016.00023

**Published:** 2016-03-08

**Authors:** Naomichi Fujiuchi, Nobuyuki Matoba, Ryo Matsuda

**Affiliations:** ^1^Department of Biological and Environmental Engineering, Graduate School of Agricultural and Life Sciences, The University of Tokyo, Tokyo, Japan; ^2^Owensboro Cancer Research Program, James Graham Brown Cancer Center, University of Louisville School of Medicine, Owensboro, KY, USA; ^3^Department of Pharmacology and Toxicology, University of Louisville School of Medicine, Louisville, KY, USA

**Keywords:** plant molecular farming, upstream production, plant biomass, plant morphology, T-DNA transfer, plant host stress responses

## Abstract

*Agrobacterium*-mediated transient expression systems enable plants to produce a wide range of recombinant proteins on a rapid timescale. To achieve economically feasible upstream production and downstream processing, two yield parameters should be considered: (1) recombinant protein content per unit biomass and (2) recombinant protein productivity per unit area–time at the end of the upstream production. Because environmental factors in the upstream production have impacts on these parameters, environment control is important to maximize the recombinant protein yield. In this review, we summarize the effects of pre- and postinoculation environmental factors in the upstream production on the yield parameters and discuss the basic concept of environment control for plant-based transient expression systems. Preinoculation environmental factors associated with planting density, light quality, and nutrient supply affect plant characteristics, such as biomass and morphology, which in turn affect recombinant protein content and productivity. Accordingly, environment control for such plant characteristics has significant implications to achieve a high yield. On the other hand, postinoculation environmental factors, such as temperature, light intensity, and humidity, have been shown to affect recombinant protein content. Considering that recombinant protein production in *Agrobacterium*-mediated transient expression systems is a result of a series of complex biological events starting from T-DNA transfer from *Agrobacterium tumefaciens* to protein biosynthesis and accumulation in leaf tissue, we propose that dynamic environment control during the postinoculation process, i.e., changing environmental conditions at an appropriate timing for each event, may be a promising approach to obtain a high yield. Detailed descriptions of plant growth conditions and careful examination of environmental effects will significantly contribute to our knowledge to stably obtain high recombinant protein content and productivity, thus enhancing the utility of plant-based transient expression systems as recombinant protein factories.

## Introduction

Plants offer several benefits for recombinant protein production, including the potential for low-cost and large-scale biomass production, the low risk of contamination with human pathogens or toxins, and the ability to perform posttranslational protein modifications (Fischer et al., 2013). Transient expression systems using *Agrobacterium tumefaciens* binary vectors enable rapid production of milligram to gram quantities of recombinant proteins in whole leafy plants, such as *Nicotiana* species (D’Aoust et al., 2010; Matoba et al., 2011; Whaley et al., 2011; Gleba et al., 2014). Pharmaceutical-grade plant-based recombinant proteins can be produced at a commercial scale in closed facilities enabling good manufacturing practice-compliant processes (Fischer et al., 2012; Warzecha, 2012). Given these benefits, transient expression systems have opened a new avenue for the production of niche biopharmaceuticals and low-cost enzymes, such as individualized vaccines (McCormick et al., 2008), emerging disease vaccines (Shoji et al., 2009; D’Aoust et al., 2010), industrial enzymes (Hwang et al., 2012), and research reagents (Pogue et al., 2010). However, transient expression systems have various challenges that are inherent to the use of whole plants. These include batch-to-batch variation of recombinant protein yields (Fischer et al., 2012; Wilken and Nikolov, 2012; Twyman et al., 2013) and unique processes to ensure effective protein extraction, recovery, and purification (Matoba et al., 2011; Buyel et al., 2015).

It is also important to achieve cost-effective upstream production and downstream processing for economic feasibility. In this context, two important yield parameters at the end of upstream production should be considered: (1) recombinant protein content per unit harvested biomass (unit: g g^−1^) and (2) recombinant protein productivity per unit area–time (unit: g m^−2^/week or month). Higher recombinant protein content is expected to reduce the cost of the downstream processing because a smaller amount of raw biomass would reduce the use of downstream consumables to obtain a given recombinant protein amount (Buyel and Fischer, 2012; Tusé et al., 2014). Meanwhile, recombinant protein productivity per unit area–time is calculated by multiplying recombinant protein content per unit harvested biomass and biomass productivity per unit area–time. Higher recombinant protein productivity can save plant growth area required to obtain a given recombinant protein amount in a given time period. This is beneficial, particularly in closed facilities with a limited batch production scale available compared to open-field production. Collectively, factors that affect recombinant protein content and productivity need to be controlled and optimized. In practice, such factors include genetic (e.g., promoter selection and codon optimization), epigenetic [e.g., posttranscriptional gene silencing suppressor, such as tomato bushy stunt virus p19 (Voinnet et al., 2003)], and environmental factors (e.g., temperature), as well as factors controlling protein stability (e.g., physiochemical/biochemical characteristics and subcellular localization) (Twyman et al., 2013). Compared to the genetic and epigenetic factors and the protein stability, less attention has been paid to the importance of environmental factors on recombinant protein content and productivity. In this review, therefore, we specifically focus on the effects of environmental factors during the upstream production on recombinant protein content and productivity in *Agrobacterium*-mediated transient expression systems.

The upstream production process may further be divided temporally and spatially into two segments with vector inoculation in between (Wirz et al., 2012; Klimyuk et al., 2014; Holtz et al., 2015): a preinoculation process for 4–5 weeks and a postinoculation process for 1–2 weeks. Following the preinoculation growth process, plants are inoculated with transgene vectors, which lead to recombinant protein accumulation during the postinoculation process. Although environmental conditions should be controlled throughout the upstream production process to obtain high recombinant protein content and productivity, optimal conditions are likely different between pre- and postinoculation processes, which may also be different from well-studied environment control for high biomass productivity per unit area–time. Environmental factors in the preinoculation process affect plant characteristics such as biomass and morphology. These plant characteristics can affect recombinant protein content and productivity. On the other hand, environmental conditions in the postinoculation process, where transgene expression takes place in plants, should be controlled for recombinant protein accumulation rather than plant growth. Accordingly, we first discuss preinoculation environmental effects on recombinant protein content and productivity in the context of plant characteristics. Then, we discuss postinoculation environmental effects on recombinant protein content and productivity from biological aspects involved in protein biosynthesis and metabolism.

## Effects of Preinoculation Environmental Factors

Plant morphology, such as the ratio of leaf weight to stem weight (leaf:stem ratio), at the time of vector inoculation can potentially affect recombinant protein content per unit harvested biomass. Stems and petioles remain uninfected if plants were infiltrated with an *A. tumefaciens* harboring a deconstructed viral vector (Gleba et al., 2014). In such cases, stems are expected to contain less recombinant protein per unit biomass than leaves. Increasing a leaf:stem ratio of plants can thus be beneficial for higher recombinant protein content per unit biomass when harvesting whole plant shoots (leaves and stems).

A lower planting density, for example, can lead to higher recombinant protein content per unit harvested biomass than a high planting density. This is because a lower planting density leads to a higher leaf:stem ratio (Poorter et al., 2012b), although it also leads to lower biomass productivity per unit area–time. Therefore, an optimal planting density should be carefully examined, considering its effect on a leaf:stem ratio and resultant recombinant protein content as well as productivity. Light environment control using artificial light sources is also important for the morphology. Using LEDs, Norikane (2015) demonstrated that *Nicotiana benthamiana* plants grown at a ratio of red-light photon flux density (PFD) to far-red-light PFD (R:FR ratio) of 0.7 had a lower leaf:stem ratio than those grown at an R:FR ratio of 1.2. Hence, a light source for the preinoculation plant growth should be carefully selected, considering its effects on a leaf:stem ratio and recombinant protein content and productivity.

We have demonstrated that optimal environmental conditions for high recombinant protein content can be different from those for high biomass productivity. When *N. benthamiana* plants were grown with nutrient solution at a higher nitrate concentration of 60 mmol L^−1^ for 2 weeks before vector inoculation, recombinant hemagglutinin content per unit leaf biomass was 1.4 times higher than that obtained from plants grown with 12 mmol L^−1^ nitrate (Fujiuchi et al., 2014). However, the higher nitrate concentration also led to the lower leaf weight per plant, which has offset the higher recombinant hemagglutinin content per leaf biomass and thus resulted in comparable recombinant hemagglutinin accumulation per plant (and hence comparable productivity per unit area–time as well) between the two nitrate concentrations. This result illustrates how a single growth factor can distinctively affect the two aforementioned yield parameters, and also point to the possibility that effects of multiple environmental factors should be evaluated for high recombinant protein content and productivity.

Despite the likely impacts of preinoculation environmental conditions on recombinant protein content and productivity, few studies have addressed the issue in detail, leaving an inconclusive assessment on this subject. More studies on preinoculation environmental effects are therefore warranted.

## Effects of Postinoculation Environmental Factors

Recombinant protein content per unit leaf biomass can be significantly altered by postinoculation environmental factors, including temperature, light intensity, and humidity. Controlling these environmental factors and maintaining appropriate environmental conditions during the postinoculation process would therefore aid in obtaining high recombinant protein content. In the subsequent sections, we summarize our current knowledge on the impacts of temperature, light intensity, and humidity during the postinoculation process on recombinant protein content.

In *Agrobacterium*-mediated transient expression systems, recombinant proteins are accumulated as a result of a series of biological events in a plant. These include T-DNA transfer from *A. tumefaciens* to the plant nuclei, transcription, translation, recombinant protein folding, assembly, maturation, and subcellular localization. In addition, plant host stress responses and protein degradation are also involved in the overall yield. Among these, we shed light on T-DNA transfer and stress responses while discussing the effects of postinoculation environmental factors, since evidence exists for the contribution of these two events to the variation of recombinant protein content at different temperatures. Given that each event has its own specific timing of effect, dynamic environment control, i.e., changing environmental conditions at an appropriate timing for each event, might help increase recombinant protein content. An example of the effectiveness of such dynamic environment control is also presented.

### Temperature

Several studies have examined the temperature dependence of recombinant protein accumulation by exposing a whole plant, a detached leaf or calli to a constant temperature during several days after infiltration or coculture with *A. tumefaciens* (Table 1). Buyel and Fischer (2012) and Buyel (2013) demonstrated the temperature dependence of the content of 2G12 (a human monoclonal anti-HIV immunoglobulin G antibody) and DsRed (a red fluorescent protein) transiently expressed in young leaf tissue of *Nicotiana tabacum*. At 6 days post inoculation (dpi), the 2G12 content at an air temperature of 21°C was 5- to 10-fold higher than those at 15 and 30°C, while the DsRed content at 25°C was considerably higher than those at 15 and 30°C. Dillen et al. (1997) demonstrated the temperature dependence of transiently expressed β-glucuronidase (GUS) in *N. tabacum* leaves, in which GUS staining of leaves at 19 and 22°C was greater than that at 15 and 25°C, while no staining was observed at 29°C, at 3 dpi. These studies, along with several others on the temperature effect on recombinant protein accumulation in leaves or callus (Kondo et al., 2000; De Clercq et al., 2002; Joh et al., 2005; Cazzonelli and Velten, 2006; Yasmin and Debener, 2010; Matsuda et al., 2012; Moon et al., 2014), suggest that recombinant protein content upon *Agrobacterium*-mediated transient expression generally has its peak at a temperature between 15 and 25°C. Although such an effect may depend on protein and/or plant species, the recombinant protein content appears to decrease sharply when the temperature becomes approximately 5°C higher or lower than the optimal temperature, indicating that temperature is an important environmental factor for high recombinant protein content. Also, these studies point to an important engineering control principle for plant-based protein factories, whereby temperature monitoring and control for a high degree of spatiotemporal uniformity in the plant growth space are necessary during the postinoculation process to avoid unexpected decrease in recombinant protein content.

**Table 1:** **Studies addressing postinoculation environmental effects on *Agrobacterium*-mediated transient expression**.

Plant species	Platform	Inoculation method	Recombinant protein	Subcellular localization	Focused environmental factor	Reference
*Nicotiana tabacum*	Whole plant	Vacuum	2G12, DsRed	Apoplast (2G12), plastids (DsRed)	Temperature	Buyel and Fischer (2012) and Buyel (2013)
*N. tabacum*	Detached leaf	Coculture	GUS	Cytosol	Temperature	Dillen et al. (1997)
*Phaseolus acutifolius*	Calli					
*Nicotiana benthamiana* *N. tabacum*	Whole plant	Syringe	Luciferase	Cytosol	Temperature, light intensity	Cazzonelli and Velten (2006)
*P. acutifolius*	Calli	Coculture	GUS	Cytosol	Temperature, light intensity	De Clercq et al. (2002)
*Lactuca sativa*	Leaf disk	Vacuum	GUS	Cytosol	Temperature, light intensity	Joh et al. (2005)
*Allium sativum*	Calli	Coculture	GUS	Cytosol	Temperature	Kondo et al. (2000)
*N. benthamiana*	Whole plant	Vacuum	Hemagglutinin	ER	Temperature, light intensity	Matsuda et al. (2012)
*N. benthamiana*	Whole plant	Syringe	Brome mosaic virus coat protein	Cytosol	Temperature, humidity	Moon et al. (2014)
Rose	Detached petal	N/A^a^	GUS	Cytosol	Temperature	Yasmin and Debener (2010)
Sunflower	Detached leaf	Vacuum	Endoglucanase, endoxylanase	Apoplast	Temperature	Jung et al. (2015)
*Arabidopsis thaliana*	Root segment	Coculture	GUS	Cytosol	Light intensity	Zambre et al. (2003)
*P. acutifolius*	Calli					
*N. benthamiana*	Detached leaf	Vacuum	Endoglucanase	Apoplast	Temperature, light intensity, humidity	McDonald et al. (2014)
*N. tabacum*	Whole plant	Coculture after spraying	GUS	Cytosol	Light intensity	Escudero and Hohn (1997)
*N. benthamiana*	Cultured cell	Coculture	GUS	Cytosol	Light intensity	Larsen (2011)
*N. benthamiana*	Whole plant	Syringe	GFP	Cytosol	Temperature, light intensity	Patil and Fauquet (2015)
*N. benthamiana*	Detached leaf	Vacuum	Alpha-1-antitrypsin	Apoplast	Humidity	Plesha (2008)
*N. benthamiana*	Detached leaf	Vacuum	Hemagglutinin	ER	Humidity	Fujiuchi et al. (2016)

*^a^Not available*.

The temperature dependence of recombinant protein content may be related to the efficiency of T-DNA transfer and plant host stress responses. The optimal temperature for T-DNA transfer from *A. tumefaciens* is reportedly 18–23°C (Atmakuri and Christie, 2008). Fullner and Nester (1996) assessed the temperature effect on T-DNA transfer. The transfer efficiency at 19°C was higher than that at 15°C, while those at 28 and 31°C were almost undetectable. This indicates that the efficiency of T-DNA transfer appears to have the temperature dependence similar to that of recombinant protein content. On the other hand, plant host stress responses can be induced at high temperatures, possibly causing leaf necrosis. The transient overexpression of the anti-HIV lectin actinohivin in the cytosol using *Agrobacterium*-mediated viral vector induced severe necrosis in *N. benthamiana* leaves at 27°C, but not at 22°C, at 4–5 dpi (Matoba et al., 2010). Similarly, we have observed necrosis in *N. benthamiana* leaves upon exposure to a postinoculation air temperature of 25°C, which was associated with low recombinant endoplasmic reticulum (ER)-targeted hemagglutinin content, while no necrosis with high hemagglutinin content was observed at 20°C (Matsuda et al., 2012). Necrosis during transient expression may be caused by a hypersensitive response and ER stress, which are likely induced by delivery of an *Agrobacterium*-mediated viral vector and an excessive abundance of misfolded proteins, respectively (Hamorsky et al., 2015). Collectively, host stress responses and necrosis induced at the higher temperature appear to be responsible, at least partly, for the lower recombinant protein content at the higher temperature.

In the postinoculation process in large-scale protein production facilities, static environmental conditions, where environmental variables are maintained almost constant, seem to be employed (Wirz et al., 2012; Holtz et al., 2015). However, air temperature may not necessarily have to be maintained constant. Jung et al. (2015) recently explored the effects of two-phase air temperature control. They observed fivefold higher content of recombinant xylanase in detached sunflower leaves treated at 20°C for the first 4 days postinoculation followed by 27°C for 2 days than that of leaves exposed to a constant temperature of 20°C. Considering that T-DNA transfer is generally completed within a single day after *A. tumefaciens* attachment to plant cells (Virts and Gelvin, 1985; Sykes and Matthysse, 1986), it may be appropriate to treat plants at a relatively low temperature, which is suitable to T-DNA transfer, during the early period of the postinoculation process. It may then be better to shift the temperature to a higher level optimal for subsequent recombinant protein accumulation and be maintained at the level for the remaining period. The result of Jung et al. (2015) illustrates the usefulness of the dynamic control of air temperature as a strategy to improve recombinant protein accumulation.

### Light Intensity

Several studies have examined the effect of light intensity during the postinoculation process on recombinant protein content (Table 1). There are reports indicating that exposure to light is essential. For example, Cazzonelli and Velten (2006) observed that the recombinant luciferase content in *N. tabacum* leaves treated in the dark was 20% of that treated at a photosynthetic PFD (PPFD) of 80–100 μmol m^−2^ s^−1^. Similarly, De Clercq et al. (2002) demonstrated that recombinant GUS was highly expressed in tepary bean callus at a PPFD of 20 μmol m^−2^ s^−1^, while almost no expression was detected in those treated in the dark. Zambre et al. (2003) supported the findings of De Clercq et al. (2002) and concluded that the negative effect of the dark treatment on transient expression was attributable to neither transcription nor posttranscriptional events but was associated with lower T-DNA transfer efficiency.

In contrast, some studies have shown that exposure to light have no positive effect on recombinant protein accumulation. McDonald et al. (2014) demonstrated that the abundance of recombinant endoglucanase in detached *N. benthamiana* leaves treated in the dark was comparable to that in leaves treated in the light. Similar results were reported for detached lettuce leaves (Joh et al., 2005), *N. tabacum* seedlings after wounding and spraying with *A. tumefaciens* suspension (Escudero and Hohn, 1997) and *N. benthamiana* cells cocultured with *A. tumefaciens* (Larsen, 2011).

Some researchers have investigated the effect of light intensity on recombinant protein content. We reported that there was no significant difference in recombinant hemagglutinin content between *N. benthamiana* plants treated at PPFDs of 100 and 300 μmol m^−2^ s^−1^ (Matsuda et al., 2012). Patil and Fauquet (2015) also reported no significant effect of PPFD on transiently expressed recombinant green fluorescent protein content in *N. benthamiana*.

Taken together, although there still is room for investigation on the necessity of lighting during the postinoculation process, one conclusion at present may be that exposure to light does not negatively affect recombinant protein accumulation. Even if exposure to light is essential, the intensity may not have to be high during the postinoculation process for recombinant protein accumulation.

### Humidity

There is limited information currently available about the effects of humidity on transient protein production in plants, as few studies have focused on this subject thus far (Table 1). Nevertheless, we have recently shown that controlling postinoculation humidity is particularly important for the detached leaf-based transient expression in *N. benthamiana*. When detached leaves are used as an expression platform, the leaves must be treated at low relative humidity for a few hours immediately after infiltration with *A. tumefaciens* suspension to remove the water occupying the intercellular space. The leaves are then incubated until harvest at high relative humidity to prevent wilting (Plesha, 2008). In this detached leaf system, we found that the removal of residual bacterial suspension water immediately after infiltration is the key factor for recombinant protein content (Fujiuchi et al., 2016). Recombinant hemagglutinin content was dramatically increased upon almost complete removal of suspension water occupying the leaf intercellular space by placing leaves at low relative humidity (approximately 25%). A similar effect might be observed when whole *N. benthamiana* plants were incubated at a high relative humidity during postinoculation process, which should be examined in future studies. Thus, the effectiveness of humidity control during the postinoculation process is worth being evaluated in whole plant-based systems.

## Conclusion and Future Directions

There is growing evidence that optimization of environmental conditions is crucial for efficient recombinant protein production in *Agrobacterium*-mediated transient expression systems. Given the significant effects of the environment on recombinant protein content, we encourage researchers in this research field to provide detailed documentation of upstream environmental conditions in their publications. For example, given the significance of postinoculation environmental factors, at least air temperature, PPFD, and relative humidity should be measured and described. Information on plant age and position of harvested leaves for protein quantification is also useful. In addition, documentations of planting density, light quality, and nutrient supply during preinoculation process are recommended. To optimize preinoculation environmental conditions, further investigations of their effects on plant morphology, i.e., biomass distribution between leaves and stem and resultant recombinant protein content and productivity are necessary. Other factors to be considered in general plant experiments are described by Poorter et al. (2012a). In addition to assessing pre- and postinoculation environmental effects individually, further studies should investigate interactive effects of multiple environmental factors to identify optimal environmental conditions for each protein of interest. Given the inherent variability of transient expression systems using whole plants, a careful experimental design encompassing a large sample size with a rigorous statistical analysis is critical to reveal the impacts of environmental factors. For production using a large number of plants in a growth facility, attention should be paid to the possible variability of each environmental factor within the plant growth space, since it may cause inconsistent recombinant protein content in individual plants and compromise batch-to-batch reproducibility. Ultimately, close monitoring and tight control of spatiotemporal environment variation will be critical to stably obtain high recombinant protein content and productivity and enhance the utility of plant-based transient expression systems as recombinant protein factories.

## Author Contributions

All authors conceived and designed the review. NF collated the literature. NF and RM wrote the manuscript. NM and RM critically revised the manuscript. All authors approved the final manuscript and agreed to be accountable for all aspects of the manuscript.

## Conflict of Interest Statement

The authors declare that the research was conducted in the absence of any commercial or financial relationships that could be construed as a potential conflict of interest.
